# Beyond Darwin: reactive heredity, burst-drift dynamics and eco‑evolutionary control of cancer

**DOI:** 10.1186/s12943-026-02683-w

**Published:** 2026-05-22

**Authors:** Laurine Lagache, Julien Salzet, Isabelle Fournier, Michel Salzet

**Affiliations:** 1 Univ. Lille, Inserm, CHU Lille, U1192, Protéomique Réponse Inflammatoire Spectrométrie de Masse (PRISM), Lille, F-59000 France; 2 Institut Universitaire de France, Ministère de L’Enseignement Supérieur, de La Recherche Et de L’Innovation, 1 Rue Descartes, PARIS CEDEX 05, 75231 France; 3 La Ligue Contre Le Cancer, Equipe Labellisée 2024, 75013 Paris, France

**Keywords:** Cancer evolution, Extrachromosomal DNA (ecDNA), Punctuated copy-number evolution, Drift-dominated interval, Whole-genome doubling, Chromothripsis, Adaptive therapy, Eco-evolutionary tumour dynamics

## Abstract

Darwinian clonal evolution provides a necessary framework for understanding tumour progression, yet emerging evidence shows that some cancers depart from a strictly gradualist tempo through episodic "macroevolutionary" leaps. We propose a unifying Burst-Drift-Control (BDC) model in which short-lived genomic reconfigurations ("bursts") that transiently alter heritable genomic states ("reactive heredity") precede longer intervals of constrained expansion, creating time-bounded vulnerabilities that clinicians may strategically steer ("control"). In this usage, the Drift component refers specifically to a drift-dominated or weak-selection interval after any initial post-burst sorting, not to the selective sweep itself. In the Burst phase, single catastrophic events, such as extrachromosomal DNA amplifications, chromothripsis, chromoplexy or whole-genome doubling, can generate major shifts in genotype and phenotype. These bursts produce new genetic configurations and can increase evolvability, for example when ecDNA circles permit oncogene dose tuning and non-Mendelian inheritance. During the drift-dominated interval, tumours may enter longer periods of constrained or near-neutral expansion in which spatial structure, clonal interference and weak or unresolved selection allow multiple lineages to persist, although the prevalence of such dynamics varies substantially by tumour type and treatment context. BDC does not assume that selective advantage is higher immediately after a burst than at later time points; later therapy or microenvironmental change can also impose strong selection. Finally, in the Control phase, if therapy is timed to exploit ecological dependencies and the fitness costs of resistance, cancer growth may be restrained as a stable, polyclonal "oligopoly" rather than progressing rapidly to an aggressive monoclonal takeover. We discuss supporting evidence, counterexamples and practical limitations, and argue that the main value of BDC lies in linking punctuated genome change to testable windows for evolutionary steering.

**Graphical Abstract:**

The Burst-Drift-Control (BDC) cycle with reactive heredity. Macro-evolutionary bursts generate heritable variation, early post-burst sorting can include strong selection, and later drift-dominated or weak-selection intervals may create opportunities for adaptive control.

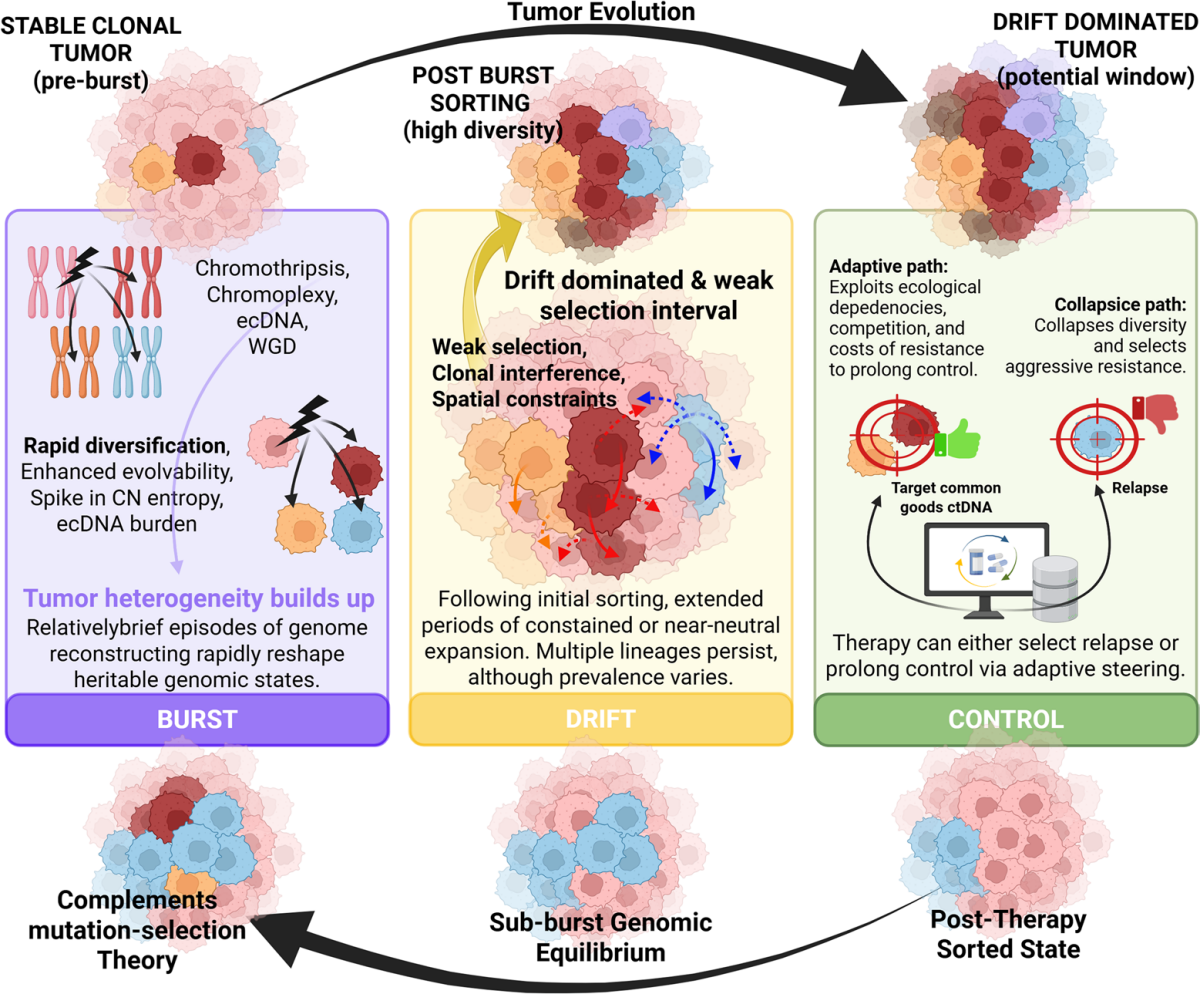

## Keypoints

- Extended Evolutionary Framework: The goal of BDC is to present a novel multifaceted framework for considering cancer development and treatment timing without contradicting conventional clonal-evolutionary analysis. It does not reject standard mutation-selection theory; rather, it emphasizes that the tempo of heritable change is often uneven and that this temporal asymmetry may have therapeutic consequences.
- Reactive Heredity & Macroevolutionary Bursts: Some tumours undergo short episodes of large-scale structural reconfiguration, including chromothripsis, chromoplexy, whole-genome doubling and ecDNA amplification. These events can rapidly expand the space of heritable phenotypes without implying that the basic cellular machinery of inheritance has been suspended.
- Drift-Dominated Expansion and Clonal Stasis: Between bursts, some tumours display prolonged intervals of constrained, near-neutral or weak-selection expansion after initial post-burst sorting. In this framework,"drift" is used in this restricted operational sense and is distinguished from the selective filtering or selective sweep that may occur immediately after a burst. BDC does not assume that selective advantage is higher immediately after a burst than at later time points.
- Therapy-Driven Evolutionary Dynamics: Relapse can emerge through several architectures, including selection of low-frequency baseline subclones, substantial clonal continuity, therapy-associated structural or mutational change, and phenotypic or epigenetic convergence. The balance among these outcomes is tumour-type dependent.
- Tumour Ecosystems and Common Goods: Tumours behave as ecological systems of interacting populations. Competition remains central, but shared growth factors, stromal support and metabolic exchange can make certain producer clones disproportionately important for whole-tumour fitness.
- Adaptive Therapy and Eco-Evolutionary Control: Proof-of-principle for adaptive therapy is strongest in selected settings such as metastatic prostate cancer. Its success is not assumed to be universal and depends on measurable tumour burden, actionable biomarkers, restartable drugs and exploitable ecological constraints on resistance.
- Clinical Outlook: The BDC model generates testable hypotheses about when genomic change is punctuated, when tumours are in relatively stable expansion phases, and when therapy modulation may outperform maximal continuous dosing. These hypotheses are intentionally framed cautiously and with clear limits of scope.

## Introduction: why Darwin is necessary but not sufficient

**Fig. 1:**
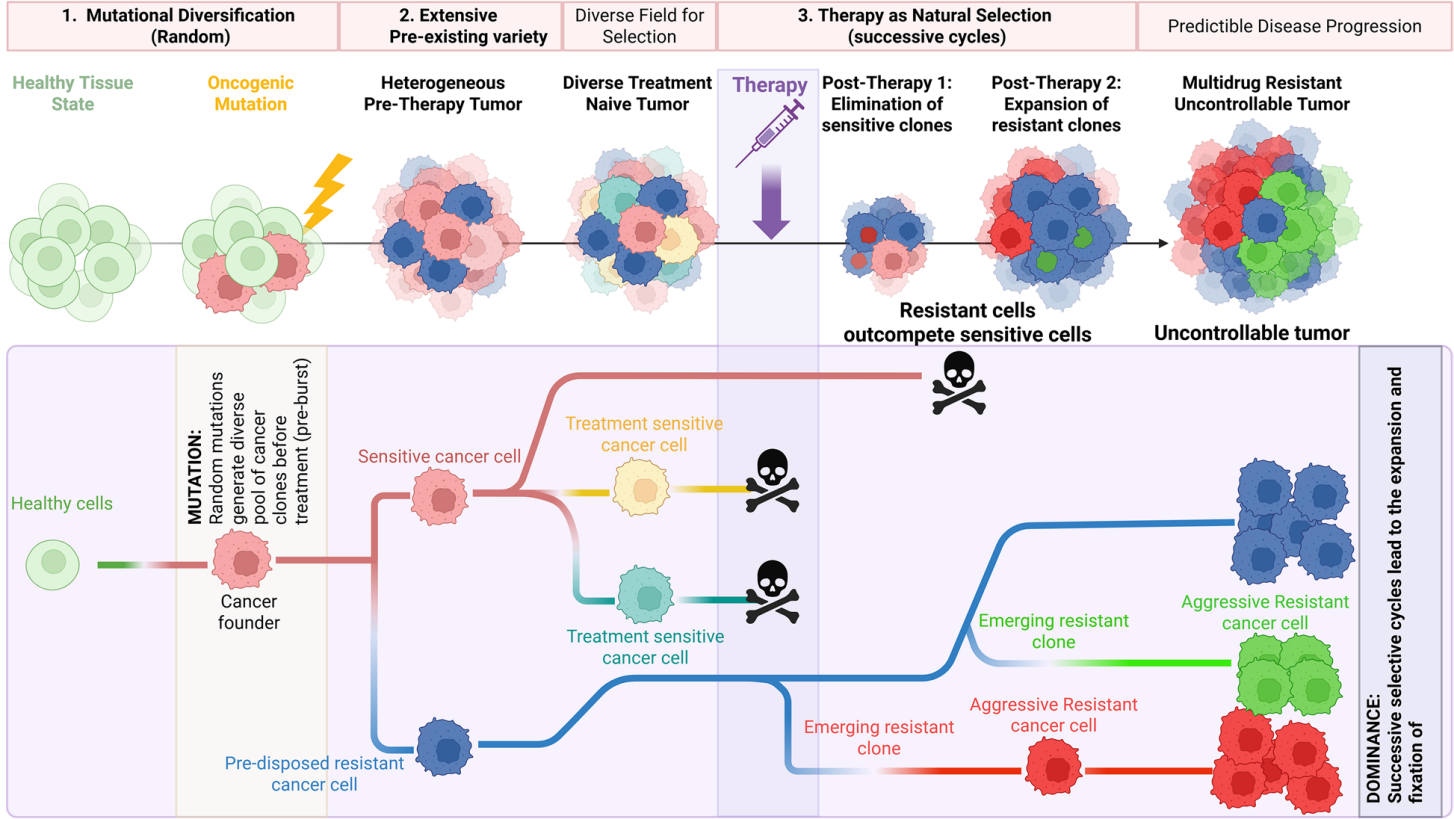
Clonal evolution through a Darwinian process of mutation and natural selection. Darwinian process in which random mutations generate diverse cancer cell clones, and therapy acts as a selective pressure. Treatment-sensitive clones are eliminated, while pre-existing or newly emerging resistant clones survive and expand. Over successive rounds of therapy, these resistant populations dominate the tumour, leading to disease progression. This evolutionary framework highlights why resistance is predictable and why strategies that anticipate or steer clonal selection are essential for long-term cancer control

In 1976, Peter Nowell first proposed that cancer progression could be explained by clonal evolution through a Darwinian process of mutation and natural selection [1]. This insight reframed tumours as dynamic, evolving populations of cells: a single transformed founder cell acquires a growth advantage, expands, and spawns diverse subclones that compete for dominance. The Darwinian paradigm has since illuminated many features of cancer biology, from the stepwise accumulation of driver mutations to the emergence of drug-resistant clones under therapy [2]. Nowell's model, often summarized as "survival of the fittest" at the cellular level, remains a cornerstone of our understanding of tumour heterogeneity and adaptation [3] (Fig. 1). Yet a strictly gradualist, gene-centric Darwinian framework does not fully capture the complex evolutionary behaviours observed in cancers.

The goal of this Perspective is to present a novel multifaceted framework for considering cancer development and therapeutic timing without contradicting conventional clonal-evolutionary analysis. BDC keeps mutation, selection and drift within standard population-genetic reasoning, but emphasizes a clinically relevant temporal asymmetry: heritable variation can sometimes be generated in short, structurally disruptive bursts; this variation can then be filtered by selection and spatial constraints; and, during later intervals of relative genomic stasis, therapy may sometimes be used to steer the tumour ecosystem rather than simply maximize cell kill. Three observations motivate this framing:

### Macroevolutionary leaps

Cancer genomes sometimes change in massive spurts rather than only through incremental alterations [4, 5]. Single catastrophic events such as chromothripsis, chromoplexy or whole-genome doubling can remodel karyotype and gene dosage in one-episode [6–9]. These events do not invalidate Darwinian selection, but they challenge strictly gradualist narratives by concentrating variation generation into short intervals. Importantly, such events can occur early, during progression, or at relapse rather than at a single privileged moment of tumorigenesis [9, 10].

### Neutral evolution and branching evolution

Many genomic changes in tumours are passengers with little detectable fitness effect, and several studies support periods of near-neutral expansion [11–13]. At the same time, neutral-like variant allele frequency spectra do not exclude selection acting below the detection limit of bulk sequencing or after a sweep has nearly completed [14]. We therefore treat neutrality as an empirical signal of weak or weakly resolved selection in a given window, not as proof that selection is absent at all later stages.

### Non-genetic and microenvironmental influences

Classical Darwinian evolution focuses on heritable genetic changes, but cancer cells also exhibit phenotypic plasticity and are profoundly influenced by the tumour microenvironment [15, 16]. The widespread presence of driver mutations in normal blood and sun-exposed skin further shows that driver acquisition alone is often insufficient for malignant outgrowth [17–19]. Whether a given alteration becomes advantageous depends on cell state, developmental context and tissue ecology, what Baggiolini and colleagues have termed oncogenic competence [20].

Taken together, these observations motivate BDC as a complementary framework for timing and interpretation. It does not replace mutation-selection theory with a separate law of tumour behaviour. Rather, BDC emphasizes an empirically recurrent temporal asymmetry: variation can be generated in concentrated bursts, filtered by selection and spatial constraints, and then reshaped again by therapy and ecology [21, 22]. Throughout, the D in BDC denotes a drift-dominated or weak-selection interval after initial sorting, not the initial selective sweep. BDC is also not expected to apply uniformly to every tumour type: some cancers are dominated by punctuated structural change, others by prolonged clonal stability, and many combine both patterns.

#### Macroevolutionary “Burst” phase: reactive heredity in cancer

A defining element of the BDC model is the Burst phase, a comparatively short interval in which the inherited genomic configuration is abruptly restructured. The cellular machinery of inheritance is not suspended; rather, the heritable substrate passed to daughter cells changes discontinuously. We use the term reactive heredity to describe this stress-associated reconfiguration of heritable genomic states, especially when structural alterations rapidly expand the space of selectable phenotypes [6, 23, 24]. Reactive heredity therefore refers to abrupt change in what is inherited, not to a violation of the basic rules by which inheritance occurs.

### Chromosomal Catastrophes (Chromothripsis and Chromoplexy)

In chromothripsis, one or a few chromosomes are fragmented and imperfectly reassembled in a single episode [5, 6]. Such events can generate large numbers of deletions, duplications and rearrangements at once, and related processes such as chromoplexy can create coordinated interchromosomal exchanges [25–28]. These catastrophes were initially emphasized as early oncogenic events, but longitudinal and advanced-disease studies now indicate that chromothriptic patterns can also persist or emerge during progression and relapse [7, 8, 10]. For BDC, the key point is therefore not that bursts are invariably early, but that structurally disruptive episodes can be temporally concentrated and evolutionarily consequential whenever they occur.

### Whole-Genome Duplication (WGD)

Duplication of the entire genome doubles chromosomal content in a single cell division error. Pan-cancer studies suggest that WGD occurs in roughly one-third of cancers overall, although prevalence varies by entity [9, 29, 30]. WGD increases gene dosage, buffers the loss of one allele, and can facilitate subsequent chromosomal instability and aneuploid evolution [9, 29, 30]. Rather than treating WGD as a universal marker of recurrence, the more defensible interpretation is that WGD can precede or accompany major clinical transitions in selected settings and is repeatedly associated with aggressive disease features and adverse outcome [9].

### Extrachromosomal DNA amplification

Another mode of burst involves oncogene amplification on extrachromosomal DNA (ecDNA) [22, 31, 32]. EcDNA is detected across many tumour types and is enriched in aggressive or advanced cancers, although prevalence estimates vary substantially by cohort and assay [32, 33]. Because ecDNAs lack centromeres, their segregation is frequently unequal, generating rapid copy-number heterogeneity across daughter cells [33–36]. Importantly, recurrent observation of specific oncogenes on ecDNA does not by itself prove biased ecDNA generation; it may instead reflect strong post-formation selection among many structurally diverse amplicons. In this sense, ecDNA illustrates how stochastic genome restructuring and subsequent selection can interact on short timescales [33, 36, 37].

These burst mechanisms share a common theme: a transient period of instability generates a large amount of heritable variation in a compressed timeframe. Subsequent selection, drift and spatial constraints determine which of those variants persist. This is why BDC is not intended as an alternative to mutation-selection theory, but as a description of how the tempo and scale of variation generation can shape later ecological and therapeutic dynamics.

A burst may also establish founder effects by generating one or more lineages with favourable ecological positioning, for example better access to nutrients, immune evasion or higher oncogene dosage, but these founder states should be inferred empirically from single-cell and multi-region data rather than anthropomorphized or assumed a priori [13, 38].

#### The drift-dominated interval: neutral evolution, weak selection and clonal stasis

Following a burst, selection can strongly filter the newly generated lineages. We do not label this initial post-burst sorting as drift. In BDC, the drift-dominated interval begins only after the major burst-generated architecture has been sorted or stabilized. During this later interval, the dominant genomic configuration may remain relatively constant while passenger diversity accumulates, spatial structure preserves multiple lineages, and selection is weak, spatially constrained or not resolvable with the available data. Thus, "drift" is used here as an operational description of observable dynamics within a time window, not as a claim that selection has ceased.

Importantly, BDC does not require that selective advantage is always higher immediately after a burst than later. Burst-generated configurations may be advantageous, neutral or deleterious depending on ecological context, and later therapy or microenvironmental change can create new selective gradients. The model instead predicts that in some tumours, temporally concentrated variation generation is followed by intervals in which the dominant architecture is stable enough to be monitored or therapeutically steered.

### Punctuated equilibrium in tumour phylogenies

Single-cell sequencing studies provide direct evidence that copy-number evolution can be temporally concentrated [13, 39]. In triple-negative breast cancer, Gao et al. found that most large copy-number alterations were acquired in one or two short bursts, followed by long periods of clonal stasis [13]. This is compatible with a drift-dominated interval, but it remains important to distinguish the initial selective filtering of burst-generated variants from the longer subsequent plateau.

### Near-neutral expansion and the Big Bang model

Deep sequencing and spatial sampling studies have shown that some tumours grow in patterns consistent with near-neutral expansion [11, 12, 40]. The Big Bang model of colorectal cancer remains an important example [12], but it does not exclude later selection. Rather, it shows that early diversification can dominate the detectable signal in bulk samples, especially when later selective events are spatially constrained or occur below the resolution limit of standard sequencing [12, 14, 41].

What counts as evidence for drift? Neutral-like variant allele frequency spectra, absence of recurrent late driver convergence, and persistent regional mosaicism can argue for weak or unresolved selection in specific windows [11, 12, 42, 43]. However, as emphasized in the debate following Williams et al., bulk whole-genome sequencing cannot reliably rule out selection among very small subclones, and false-neutral classifications are possible when an expanding subclone lies outside the measurable regime [14]. We therefore interpret drift as a quantitatively limited claim about observable dynamics, not as evidence that selection has ceased altogether.

**Fig. 2:**
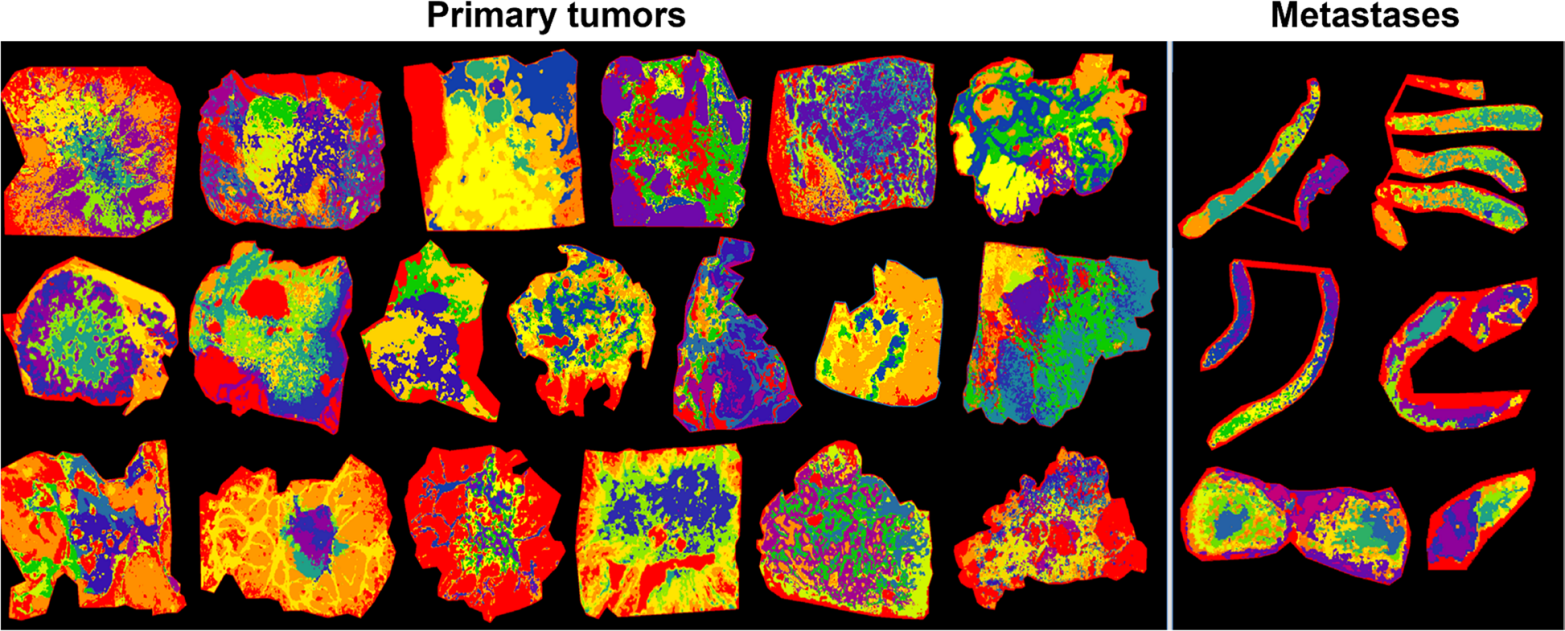
Longitudinal studies of Luminal breast cancer from primary tumour to metastases analyzed by MALDI mass spectrometry imaging and segmented using *k*-means analyses [49, 50].

Tumour-type heterogeneity is central here. Several paired-sample studies in glioblastoma and AML report substantial clonal stability or mutational continuity across relapse, whereas others show divergent evolution or non-genetic convergence [44–47]. Likewise, drug resistance can arise through genetic change, phenotypic plasticity or convergent epigenetic states rather than through a single new resistant genotype [15, 47, 48]. Our own work in luminal breast cancer supports persistent phenotypic heterogeneity over time, and these observations should be interpreted as context-dependent rather than universal [49, 50] (Fig. 2).

**Fig. 3:**
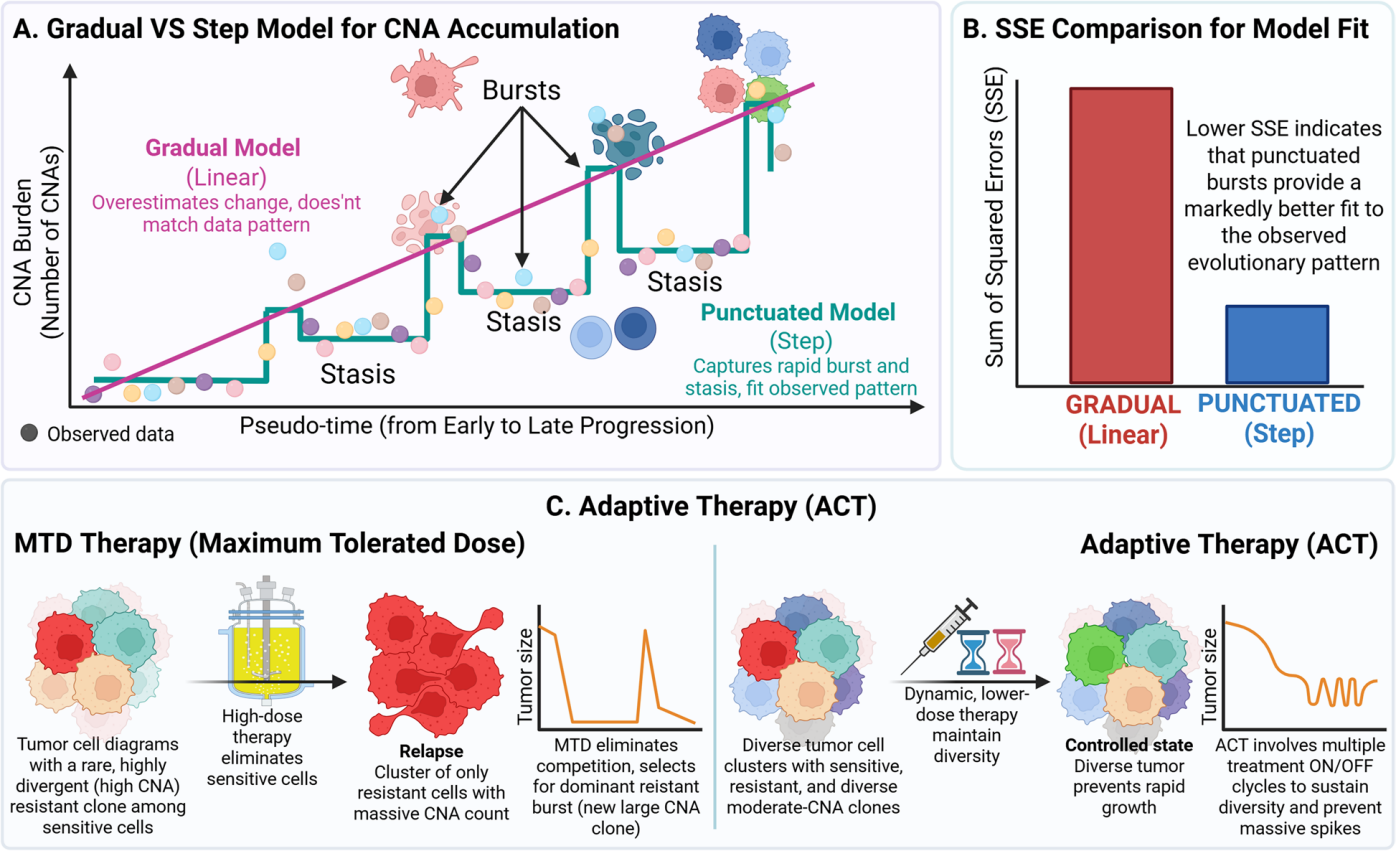
Stylized comparison of punctuated copy-number dynamics and adaptive therapeutic control. **A** A conceptual comparison of gradual (linear) versus punctuated (step-like) models for copy-number alteration (CNA) burden over evolutionary pseudo-time. The punctuated model illustrates rapid generation of copy-number diversity followed by a period of relative architectural stasis; it should not be interpreted as showing that selection is absent, but rather that any subsequent selection may be weak, spatially constrained or not resolved by the available data. **B** Model fit can be compared using the sum of squared errors (SSE) in datasets where serial or single-cell measurements are available. **C** A schematic contrast of tumour dynamics under maximum tolerated dose (MTD) therapy versus adaptive therapy (ACT). MTD may eliminate sensitive cells and thereby release resistant populations from competition, whereas ACT aims to preserve exploitable competition by dynamically modulating treatment. This panel is intended as a conceptual model rather than a universal prediction for every tumour type

In summary, the drift-dominated interval describes a spectrum of post-burst dynamics ranging from near-neutral expansion to weak-selection or spatially structured stasis. It does not imply that random drift alone explains every increase in clone size. The practical value of the concept is that it highlights intervals in which the tumour may be growing without obviously reorganizing its dominant genomic architecture, thereby creating windows for monitoring or ecological intervention (Fig. 3).

#### Therapy triggers new bursts and reshapes clonal composition

Therapy is one of the strongest selective forces a tumour encounters, but cells that survive initial treatment are not necessarily the fittest in any deterministic sense. Survival may reflect pre-existing resistance, drug-tolerant persistence, microenvironmental sanctuary, or stochastic escape [15, 44, 48]. What therapy reliably does is reshape the fitness landscape. In some settings it selects low-frequency resistant clones; in others it leaves much of the original architecture intact; and in others still it is followed by phenotypic or epigenetic convergence rather than a single genetically dominant escape lineage [44–47].

A clear illustration of divergent selection comes from medulloblastoma. Morrissy et al. sequenced paired diagnosis and relapse samples and showed that recurrent disease was often dominated by lineages that were minor at diagnosis, with limited overlap between primary and relapse mutation sets [51]. This example is retained because it strongly supports the existence of therapy-shaped evolutionary bottlenecks, but it should not be generalized to all tumour types.

Across adult cancers, longitudinal studies reveal several relapse architectures rather than one. In glioblastoma, recurrences can be linear, divergent or genomically stable depending on the case [44–46]. In AML, a substantial fraction of relapses occur without new driver mutations and instead show convergent epigenetic evolution [47]. These counterexamples are important because they delimit the scope of BDC: relapse is not always the outgrowth of a hidden genetically resistant subclone, even though that scenario clearly occurs in some diseases such as medulloblastoma, *EGFR*-mutant lung cancer and colorectal cancer treated with *EGFR* blockade [51–54].

Therapy can also induce stress responses that drive genetic instability, but the biological and clinical consequences are context dependent. This is one possible route to relapse, not a universal rule.

Certain chemotherapies and radiation can leave surviving cancer cells with new mutational or structural lesions [55, 56]. Temozolomide-associated hypermutation in glioblastoma is a good example: it can generate large numbers of new mutations, yet recent longitudinal analyses suggest that hypermutated recurrences may also be associated with longer time to recurrence and improved overall survival in some patients rather than with a simple "more aggressive clone" narrative [46, 55].

Some targeted therapies may also select or induce non-genetic states that later enable adaptation. Drug-tolerant persisters can survive initial exposure through reversible chromatin and cell-state programs and then acquire or reveal additional genetic diversity over time [15, 48, 57, 58]. In this setting, therapy-driven evolution may proceed through phenotypic convergence first and genetic diversification later, rather than through an immediate structural burst.

More broadly, treatment need not accelerate tumour evolution primarily by increasing mutation rate. The abundance of driver mutations in normal tissues and clonal hematopoiesis argues that driver acquisition alone is often not rate-limiting [17–19]. Treatment can instead act by selecting among pre-existing genetic diversity, exposing ecological dependencies, or stabilizing drug-tolerant phenotypic states.

From a BDC standpoint, therapy can therefore do at least three things: select pre-existing lineages, precipitate new structural or mutational change, or canalize phenotypic convergence. The control problem in oncology is to distinguish among these possibilities early enough to adapt treatment before one trajectory becomes clinically dominant.

#### Tumour ecosystems and common goods

**Fig. 4:**
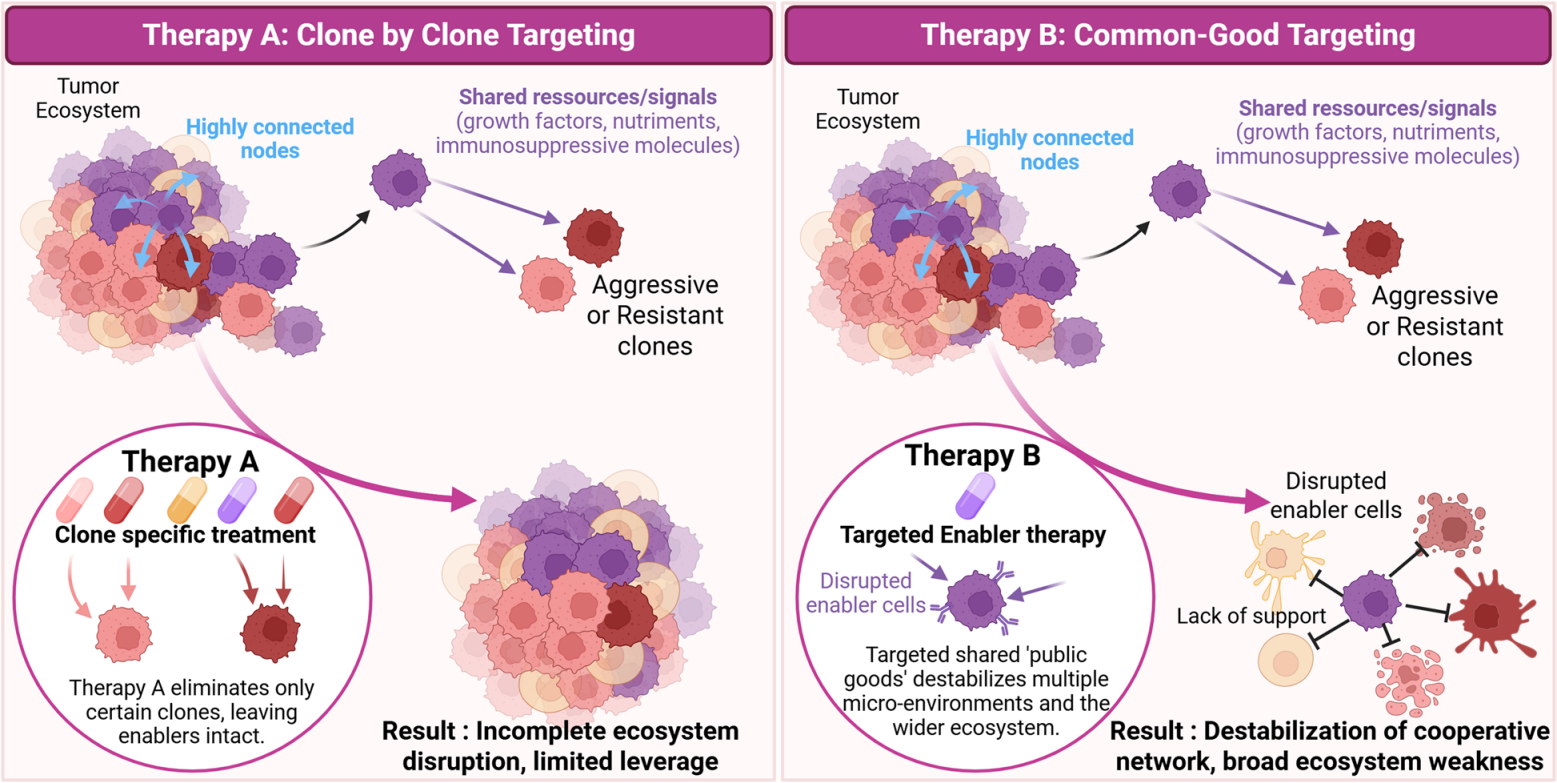
Targeting “Common-Good” Cancer Cells to Disrupt Tumour Ecosystems. This figure illustrates how tumours behave as complex ecosystems composed of diverse cancer cell subpopulations that interact with each other and their surrounding microenvironments. Some cancer cells, highly connected nodes, produce shared resources or signals that benefit the entire tumour community. These common-good enablers allow other, more aggressive or therapy-resistant clones to survive and proliferate. While traditional treatments (Therapy **A**) may eliminate only certain clones, targeting these key enablers (Therapy **B**) can destabilize cooperative networks across multiple microenvironments. By disrupting the shared “public goods” that sustain tumour growth, this strategy may weaken the wider tumour ecosystem and provide broader leverage than clone-by-clone targeting

A tumour is not just a clone of malignant cells; it is a complex community of diverse cell populations, cancer cells of various subclones, as well as non-cancerous stromal cells, immune infiltrates, etc.… The interactions among these players can significantly influence tumour growth and evolution [59]. Increasingly, cancer is viewed through an ecological lens: a tumour as an evolving ecosystem. In ecological terms, tumour cells can engage in competition, predation (for example, immune cells killing cancer cells), symbiosis, and parasitism with one another and with the host environment. These interactions create selection pressures and cooperative opportunities that are absent in a single-cell, gene-centric perspective [1–4]. One particularly important concept in tumour ecology is the existence of “common goods” and “common-good producers” (sometimes referred to colloquially as “common gooder” clones). Common goods are factors produced by some cells that can be utilized by others in the tumour microenvironment (Fig. 4). They can be:

### Growth factors and cytokines

For example, a subclone may produce high levels of a growth factor like VEGF (vascular endothelial growth factor) to stimulate angiogenesis. All cells in its vicinity benefit from the resulting new blood vessels, not just the producer. Another classic example is IL-6 or other inflammatory cytokines produced by some subsets of cells that create a pro-tumour immune environment that benefits neighboring cancer cells [1, 5, 6].

### Extracellular matrix components

Some cancer (or stromal) cells secrete matrix proteins or remodeling enzymes that create tracks for invasion. Neighboring clones that don’t produce these themselves can still exploit the remodeled matrix to invade or metastasize [7, 60].

### Metabolic public goods

Tumour metabolism often involves cooperation. A famous case is the lactate shuttle: some cancer cells (especially in hypoxic regions) perform glycolysis and secrete lactate, which can then be taken up by other cancer cells (in more oxygenated regions) as a fuel for oxidative phosphorylation. Here, lactate is a common good that allows resource sharing. Similarly, some subclones might secrete glutamine or other metabolites that feed their neighbors [8, 9].

### Niche construction

Subclones might modify the local environment in ways that make it more habitable for all. For instance, cancer-associated fibroblasts (not cancer cells themselves, but part of the ecosystem) secrete growth factors and chemokines that benefit cancer cells broadly; a particular cancer subclone could “recruit” or activate fibroblasts to do this job, indirectly sustaining other clones too [10].

The presence of common goods means that not all cancer clones are acting as isolated competitors; some are effectively cooperating (even if unintentionally) by supporting shared resources (Fig. 4). There may exist “keystone” clones in a tumour, analogous to keystone species in an ecosystem, whose activities disproportionately enable the survival and proliferation of the whole community. A clone producing a growth-essential common good is one such keystone. This has profound therapy implications: targeting a keystone common-good producer might collapse the entire ecosystem’s growth, whereas targeting a “selfish” clone that only contributes to its own growth might yield a much more limited effect. Tabassum and Polyak (2015) illustrated this concept vividly [2]. In their framework (Fig. 4, selfish target), if you eliminate a subclone that does not provide any benefit to others (i.e., a pure competitor), the rest of the tumour those “neutral” or other competing clones can continue growing unabated. The initial kill might shrink the tumour a bit, but ultimately some other clone will fill the void, and the tumour may soon resume progression (relapse). If instead you eliminate a subclone that was providing a crucial factor (say a diffusible growth promoter that all clones need), the overall tumour growth slows or stops (Fig. 4, common-good target). Even clones that are genetically untouched by the drug feel the impact because their support system is gone. This could translate to a much more durable response or even long-term control, as no other clone can easily replace that function (unless a rare one can also produce the factor) [2, 11].

There are examples of this principle in action. One emerging area is targeting “niche” factors in the microenvironment. For instance, some subclones or stromal cells produce hepatocyte growth factor (HGF) which can make tumour cells resistant to certain therapies (like RAF inhibitors in melanoma via paracrine activation of MET) [11, 12, 61]. Inhibiting HGF or its receptor can remove that protective common good, resensitizing the tumour. Another example is in multiple myeloma, where the cancer cells depend on interleukin-6 from the bone marrow stroma; trials of IL-6 inhibitors have been explored to cut off this support [13]. While not a subclone difference, it’s the same concept of removing a shared resource. We can also consider immune evasion as a kind of common good. If one subclone expresses a factor that strongly suppresses T cell activity (like PD-L1 or secreting TGF-β), it might protect not only itself but neighboring antigenic clones from immune attack [14, 15]. Eradicating that immune-evasive subclone or blocking its immune-suppressive output could expose the entire tumour to immune clearance. Now, how do we identify these “common gooder” clones? This remains challenging. It might involve spatial profiling to see which regions/clones express certain factors, and correlating that with proliferative indices in neighboring cells. Transcriptomic analyses can highlight subpopulations with high expression of secreted factors. Recent single-cell multi-omics and spatial transcriptomics technologies are helping map these interactions [16–18]. As one example, Marusyk et al. (2014) [6] showed that injecting mixtures of breast cancer cell line subclones into mice yielded faster tumour growth than clones alone, because one subclone secreted IL-11 that enhanced the proliferation of another subclone. Such experiments indicate that cooperation is real and can be exploited [62–64].

The tumour ecosystem concept also includes negative interactions like competition (Fig. 4). Clonal competition is the default assumption in cancer, “survival of the fittest”, and indeed occurs (for example, nutrient competition, competition for space) [3, 4, 19]. Clonal interference, as mentioned, can slow evolution because multiple clones with similar fitness keep each other in check. There’s also the possibility of amensalism (one clone harms another without itself benefiting, say by secreting waste or toxins that kill nearby cells) and commensalism (one benefits, the other is neutral). All these dynamics mean that removing or adding a clone (as therapy does) can have ripple effects. A fascinating implication is that the composition of clones in a tumour could determine its aggressiveness more than any single clone’s properties. For instance, one might find that a tumour containing a mix of clones that each specialize (metabolically or signaling-wise) grows faster than a monoclonal tumour, because the clones complement each other’s needs, a division of labor scenario. Conversely, a highly heterogeneous tumour might also be less “efficient” if the clones are at odds (lots of competition causing a sort of stalemate). There’s evidence for both scenarios in different contexts. From a treatment perspective, an eco-evolutionary view leads to strategies that manipulate interactions [20, 23]:

Thus, recognizing that a tumour is not homogeneous but rather an ecosystem suggests that the ecosystem could be steered by removing certain elements or by tilting competitive balances. Traditional therapy has often tried to remove as many tumour cells as possible, which can backfire by removing suppressive interactions as well. In contrast, an ecological therapy approach might intentionally spare some populations or target specific non-lethal vulnerabilities that weaken the whole system. A concrete example is the concept of “sucker’s gambit” therapy: encourage the growth of a less harmful clone (perhaps by not treating it) while specifically suppressing a more dangerous one, essentially managing competition to keep the tumour in a less aggressive state.

In summary, tumours operate as multi-species systems with emergent behaviours that cannot be predicted by studying a single clone in isolation. Some subclones play outsized roles in supporting tumour growth through common goods, and their presence or absence can alter disease trajectory. By thinking ecologically, oncologists can identify intervention points such as shared growth factors, stromal dependencies, or immune-suppressive niches whose disruption may have tumour-wide effects. This systems perspective motivates adaptive or evolutionarily informed therapy, which seeks to manage competition and cooperation rather than simply maximize immediate cell kill.

#### Adaptive therapy: containment rather than maximal eradication

Conventional oncology has long pursued a cure-oriented paradigm: hit the tumour with the maximum tolerated dose of therapy to eliminate as many cells as possible. However, such aggressive strategies can also accelerate the emergence of resistance. The Control phase of BDC asks whether, in selected contexts, better outcomes may come from modulating rather than maximizing therapy. Adaptive therapy is the clearest example. Its logic is ecological: benefit is greatest when treatment response can be monitored, when resistant and sensitive populations interact competitively, and when resistance carries some ecological disadvantage or constraint. We therefore no longer present adaptive therapy as universally applicable.

- Treatment is initiated and reduces tumour burden, typically affecting sensitive populations more strongly than resistant or tolerant ones.
- Instead of continuing until disease is driven to its minimum detectable level, treatment is held or reduced once a predefined biomarker or tumour-burden threshold is reached.
- Off-treatment intervals allow sensitive cells to recover and continue suppressing resistant populations; this does not require strict equality of drug-free fitness, only that competitive interactions remain exploitable.
- Therapy is resumed when burden rebounds to a second threshold, creating a feedback cycle intended to delay competitive release rather than eliminate every last sensitive cell.

This cycle aims for controlled oscillation rather than unchecked outgrowth of a resistant clone. Preclinical and early clinical studies support the plausibility of this approach but also define its limits. In breast cancer xenografts, adaptive paclitaxel maintained longer control with less cumulative drug than standard high-dose scheduling [65]. In metastatic castration-resistant prostate cancer, pilot studies of adaptive abiraterone reported longer time to progression and lower cumulative drug exposure than contemporaneous standard dosing controls [66]. These results are encouraging proof-of-principle rather than definitive evidence of broad generalizability.

**Fig. 5:**
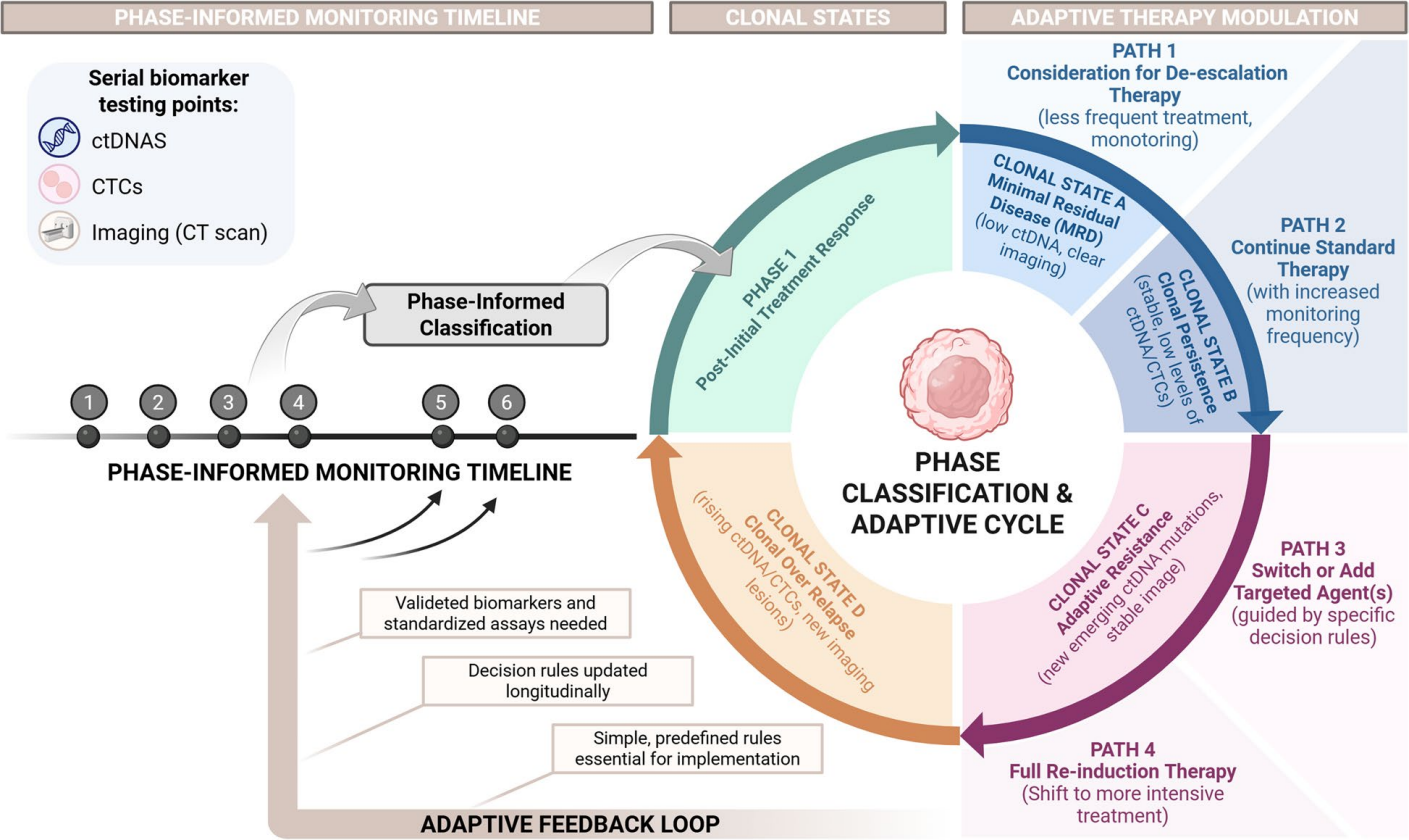
Conceptual scheme for phase-informed monitoring and adaptive therapy. This schematic illustrates how serial biomarkers, for example ctDNA, circulating tumour cells and imaging, could in principle help guide therapy modulation in selected settings. It is intended as a conceptual research roadmap rather than a representation of current routine clinical practice; reliable implementation would require validated biomarkers, standardized assays and simple decision rules that can be updated longitudinally

Adaptive therapy is most realistic in diseases with a tractable biomarker (for example PSA, ctDNA or a reproducible imaging measure), drugs that can be stopped and restarted safely, and a mathematical model simple enough to be updated from longitudinal data. Clinically deployed approaches have mostly used low-dimensional ecological models or threshold rules rather than exact reconstruction of every clone [65–67]. In many cancers, the necessary biomarkers, drug repertoires or scheduling flexibility are not yet available (Fig. 5).

It is important to emphasize that the success of adaptive therapy hinges on the assumption that sensitive and resistant populations compete, and that resistant cells pay a fitness cost in the absence of drug. This cost may not be present in all tumours or for all resistance mechanisms; therefore, adaptive therapy is not a one-size-fits-all solution and requires empirical validation in each context.

#### Ecological targeting: hitting the tumour’s weak links

Another facet of the Control phase is ecological targeting based on shared vulnerabilities. If tumour growth depends disproportionately on common-good producers or other highly connected ecological functions, then intervening at those nodes may have broader effects than targeting a single fast-growing clone. At present, however, such strategies remain largely preclinical or theoretical, and the relevant dependencies must be demonstrated longitudinally rather than assumed from static profiling alone [68–70]. We therefore frame ecological targeting as a promising but still exploratory component of BDC, not as a clinically established programme.

In summary, the Control phase treats therapy as an attempt to steer tumour composition with measured interventions rather than always pursue eradication at any cost. By harnessing competition, cooperation and the tumour’s ecological structure, control strategies may prolong benefit in selected settings, but they require careful monitoring and remain more mature in some cancers than in others. The next section outlines cautious, testable predictions rather than deterministic claims.

#### Predictions and distinguishing features of the BDC model

The Burst-Drift-Control framework makes several experimentally testable hypotheses, but these should be treated as non-exclusive signatures rather than all-or-none predictions (Fig. 3). Some tumours may exhibit only one cycle, others several, and many may show mixtures of punctuated change and continuous selection.

### Punctuated genomic changes

Measures of structural complexity such as copy-number burden, copy-number entropy or ecDNA content may change discontinuously in some tumours rather than accumulating smoothly over time [13, 33, 36, 51]. The prediction is not that every cancer evolves in steps, but that step-like transitions should be detectable in at least a meaningful subset of cases.

### Phenotypic transitions tied to bursts

Major clinical or histologic shifts may sometimes follow structural reconfiguration, but this should be treated as a longitudinally testable hypothesis rather than a rule. In some cancers, phenotypic transitions may instead reflect epigenetic or cell-state convergence without large new genetic events [46, 47].

### Long stasis with hidden selection caveats

If a tumour is in a drift-dominated interval, serial sampling should show limited detectable change in the dominant genomic architecture. However, this must be interpreted cautiously because apparent neutrality in bulk data can coexist with hidden selection among small clones, spatially restricted sweeps or later therapy-induced selective gradients [11, 12, 14]^.^

### Polyclonal control versus monoclonal relapse

Successful adaptive control is predicted to preserve mixed populations for longer, whereas continuous maximal therapy often culminates in resistant dominance [65, 66]. This prediction is directly testable in trials that combine serial biomarkers with evolutionary modelling.

### Outcome improvements in selected settings

Treatment protocols explicitly designed around competition, ecological dependencies and feedback dosing should improve time to progression only where the underlying assumptions are met. BDC therefore predicts benefit in a subset of patients, not in every tumour indiscriminately [65–67].

### Dynamic biomarkers

ctDNA, multiregion sequencing, single-cell measurements and spatial imaging may reveal stepwise changes before overt clinical transition [71–73]. Whether copy-number entropy, ecDNA abundance or lineage-state markers can be used prospectively as phase indicators remains an open but testable question.

Therapy timing matters: If bursts and drift-dominated intervals can be detected with sufficient confidence, then timing therapy relative to evolutionary phase should influence outcome. At present this remains more a modelling and monitoring challenge than a routine clinical capability.

A distinctive feature of BDC is that it treats clinical intervention as part of the evolutionary cycle rather than as an external afterthought. The framework is therefore most useful when it helps explain why different dosing logics, monitoring intensity or ecological targets might produce different evolutionary futures from the same starting disease.

In summary, the BDC framework proposes measurable footprints—abrupt structural reconfiguration, intervals of constrained expansion, and control strategies aimed at preserving competition, while explicitly acknowledging that none of these features is expected to dominate every cancer.

#### Monitoring and intervening in tumour evolution: tools and future directions

Realizing the potential of the BDC model in clinical practice depends on repeated measurement rather than narrative intuition. Table 1 summarizes the relevant toolkit. Single-cell and multi-region sequencing can detect punctuated structural change; liquid biopsy can track emerging resistance and shifting clonal mixtures; spatial transcriptomics and proteomics can identify ecological dependencies; and low-dimensional eco-evolutionary models can translate serial measurements into practical dosing rules. At present, the major limitation is not a complete lack of data but the difficulty of collecting data at sufficient frequency, standardizing assays across sites, and mapping those measurements to simple decisions. For that reason, the most realistic near-term applications are in diseases with accessible biomarkers and restartable therapies, whereas full closed-loop control across most solid tumours should still be regarded as aspirational.

**Table 1:** Emerging tools to track and steer tumour evolution

Method Category	Techniques and Examples	Utility for BDC Dynamics
*Genomic Profiling*	- **Bulk Next-Generation Sequencing (NGS):** Whole-exome or genome sequencing of tumour samples - **Multi-region sequencing:** Sampling spatially separated tumour sectors - **Longitudinal bulk sequencing:** Sequencing serial biopsies	Identifies subclonal mutations and clonal architectures. Multi-region or serial sequencing can reveal branched vs. linear evolution, detect emerging driver clones, and measure neutral evolution (e.g., via mutation allele frequency distributions). Provides baseline evidence of bursts (e.g., chromothripsis signatures) and drift (many passengers, power-law mutation frequencies)
*Single-Cell Genomics*	- **Single-cell DNA sequencing:** Copy-number and mutation profiles of individual cells - **Single-cell RNA sequencing:** Transcriptomic states of individual cells .- **Single-cell phylogenetics and lineage tracing:** Reconstructing trees from single-cell data; CRISPR barcoding in model systems	Distinguishes punctuated vs. gradual evolution by directly observing if cells cluster into discrete genomic subclones. Single-cell DNA-seq can detect if CNAs occurred all at once (burst) or sequentially. RNA-seq can identify coexisting cell states (including drug-tolerant persisters) and monitor phenotypic drift. Lineage tracing experiments in animals or organoids can experimentally validate burst–drift patterns and test adaptive therapy in controlled settings
*Liquid Biopsies*	- **Circulating tumour DNA (ctDNA) sequencing:** Detecting tumour-derived DNA mutations in blood - **Circulating tumour cells (CTCs):** Isolation and genomic/phenotypic analysis of intact tumour cells in blood - **Exosomal and proteomic assays:** Tumour-derived vesicles/proteins in blood	Provides a minimally invasive, **real-time readout** of tumour evolution across the whole body. ctDNA can track mutation allele frequencies over time, revealing emerging resistant clones months before clinical progression. It can also measure overall heterogeneity (e.g., via the number of variants or fragmentation patterns). CTCs allow functional studies on live cells: one can test drug sensitivity or identify epithelial–mesenchymal transition states in CTCs (indicating adaptive responses). Liquid biopsies are critical for adaptive therapy feedback loops, enabling clinicians to adjust treatment on-the-fly based on evolutionary trends (e.g., rising ctDNA of a known resistant mutation prompting a switch in drugs)
*Spatial and Single-Cell Imaging*	- **Spatial transcriptomics & proteomics:** High-plex RNA/protein mapping on tumour sections (e.g., 10 × Visium, CODEX, IMC) - **Multiphoton/optical imaging of tumours:** Visualizing clonal growth in situ in model systems (e.g., confetti mouse models with multicolor clones) - **Digital pathology with AI:** Quantifying spatial heterogeneity in histology slides	Maps the tumour **ecosystem** in space. Can identify niches (hypoxic vs normoxic regions, invasive fronts) and how different clones localize to them. For example, spatial transcriptomics might show that a “common-good” IL-6 producing clone is situated in the tumour core and surrounded by high-proliferation neighbors, suggesting an ecological interaction to target. Imaging can also show clonal intermixing or segregation, informing whether drift is happening neutrally (random mix) or selection has created regional clonal sweeps. These methods help pinpoint targets for ecological therapies, e.g., if a certain microenvironmental factor is sustaining multiple clones, we can see it spatially and plan to target it
*Mathematical & Computational Modeling*	- **Evolutionary dynamics modeling:** Agent-based or differential equation models incorporating mutation, selection, drift (e.g., cellular automata of tumour with therapy on/off cycles) - **Predictive simulations for adaptive therapy:** Patient-specific modeling using initial tumour parameters to simulate different dosing strategies - **Bioinformatics tools for neutrality and selection:** e.g., dN/dS ratio tests, mutant allele distribution analysis; algorithms to detect punctuated evolution from genomic data	Serves as a **“virtual laboratory”** to test and optimize control strategies. Models can predict when a tumour will escape control and help design adaptive protocols (e.g., what PSA threshold to use, or what drug rotation might work best). They also help interpret data: e.g., a power-law VAF distribution can be checked against a neutral model to confirm drift. By integrating patient-specific data (growth rates, carrying capacity, sensitivity), models can be used prospectively to guide therapy-essentially providing the “phase estimation and policy outputs” envisioned in a closed-loop adaptive therapy system. Over time, as we gather more outcomes, machine learning could also assist in predicting which tumours are likely to follow BDC dynamics and thus benefit most from adaptive approaches

## Conclusion, scope and future directions

Cancer's ability to evolve is a major reason it remains lethal. The classical Darwinian view explained much of this behaviour, but many tumours also show punctuated structural change, prolonged periods of constrained expansion, and strong ecological dependencies. The BDC model is offered as a synthesis of these observations and as a hypothesis-generating, clinically oriented lens for deciding when to intensify, when to monitor, and when to modulate treatment in selected contexts. BDC is therefore best understood as context-dependent and complementary to standard population genetics.

Its limitations should be stated plainly. BDC is not a universal law, not a formal model with fixed phase boundaries, and not a claim that every tumour alternates cleanly between burst, post-burst sorting, drift-dominated and control states. In many cancers, selection may remain continuous, phenotypic convergence may dominate over genetic divergence, and available data may be insufficient to distinguish neutrality from hidden subclonal selection.

Several specific directions nevertheless follow from this framework, each of which will require prospective validation rather than conceptual enthusiasm alone:

Integrated diagnostics in practice: Serial ctDNA, imaging and selected tissue sampling may eventually allow clinicians to distinguish stable, shifting and burst-prone disease states, but only if assays are standardized and actionable thresholds are validated.

New therapeutic targets from eco-evolutionary insights: Common-good producers, ecDNA dependencies and lineage-specific constraints may provide tumour-wide leverage points that are missed by purely clone-centric strategies.

Patient-tailored evolutionary strategies: Some tumours will remain best approached with curative intent, whereas others may benefit more from containment, sequencing of drugs, or controlled intermittent therapy. An evolutionary framework is most useful when it sharpens this distinction rather than replacing clinical judgement.

Ultimately, the practical goal is not coexistence for its own sake but delayed lethal progression and preserved quality of life when eradication is unrealistic. In some settings, that may mean steering disease into a more indolent state; in others, it will mean recognizing when aggressive elimination remains the better strategy.

In conclusion, "beyond Darwin" does not mean against Darwin. It means combining selection with punctuated structural change, ecological context and therapy-aware control. The BDC framework is most useful when it sharpens questions of timing, heterogeneity and intervention in tumours where these dynamics are measurable, rather than when it is treated as a universal template for all cancers.

As studies of cooperation among tumour cells have shown, tumour behaviour can reflect collective properties that are not reducible to isolated clones [74]. In cancer, the interactions among clones and with the microenvironment create emergent properties that can be exploited therapeutically. [75].

## Data Availability

No datasets were generated or analysed during the current study.
